# A Pilot Feasibility Study of Mindful Walking in Older Adults: Exploratory Bayesian Estimates of Psychological Distress and Alexithymia

**DOI:** 10.3390/ijerph23070836

**Published:** 2026-06-25

**Authors:** Alessandro Germani, Antonella Lopez, Claudia Mirenghi, Manuela Nicoletta Di Masi, Andrea Bosco

**Affiliations:** 1 Faculty of Law, Giustino Fortunato University, 82100 Benevento, BN, Italy; a.germani@unifortunato.eu; 2 Department of Humanities, Social Sciences, and Education, University of Molise, 86100 Campobasso, CB, Italy; 3 Department of Educational Sciences, Psychology, Communication, University of Bari, 70121 Bari, BA, Italy; c.mirenghi@phd.uniba.it (C.M.); andrea.bosco@uniba.it (A.B.); 4 IBiSS Società Cooperativa Sociale, 70018 Rutigliano, BA, Italy; presidente@ibiss.org

**Keywords:** mindfulness, mindful walking, Bayesian modeling, pilot feasibility study, older adults

## Abstract

**Highlights:**

**Public health relevance—How does this work relate to a public health issue?**

- Psychological distress, loneliness, somatic symptoms, and reduced well-being are common public health concerns in older adults.
- Mindful walking offers an accessible mind–body approach combining physical activity and mindfulness for healthy aging.

**Public health significance—Why is this work of significance to public health?**

- The intervention showed high feasibility, adherence, acceptability, and tolerability in community-dwelling older adults.
- Exploratory findings suggested improvements in depressive symptoms, somatic complaints, alexithymia, mindfulness, and psychological quality of life.

**Public health implications—What are the key implications or messages for practitioners, policy makers and/or researchers in public health?**

- Mindful walking may be a low-cost, scalable intervention suitable for community and health-promotion programs for older adults.
- Future controlled trials are needed to confirm efficacy, test mechanisms, and guide implementation in public health settings.

**Abstract:**

Population aging demands accessible interventions for psychological well-being in later life. This work evaluated the feasibility and acceptability of an 8-week mindful walking program in community-dwelling older adults and generated exploratory estimates of within-person change across emotional, psychosomatic, and psychological outcomes. Thirteen community-dwelling older adults participated in a pilot human trial with assessments at baseline, post-intervention, and one-month follow-up. Measures included depression, anxiety, somatic symptoms, mindfulness, mind wandering, alexithymia, quality of life, and attachment style. Primary feasibility outcomes indicated high acceptability and participant satisfaction, good physiological tolerance and full adherence. Secondary exploratory analyses suggested within-person reductions in depressive symptoms and alexithymia, while somatic symptoms decreased notably by follow-up. Mindfulness increased and was maintained over time, while mind wandering displayed a probable long-term decrease. Psychological quality of life improved and remained elevated, whereas physical, social, and environmental quality-of-life domains showed uncertain trends. Trait anxiety decreased post-intervention but returned toward baseline at follow-up, while state anxiety and attachment styles remained stable. Within pilot design limits, mindful walking may be a feasible intervention for older adults, associated with exploratory within-person patterns suggesting possible improvements in certain psychological outcomes, which should be interpreted as preliminary and descriptive signals pending confirmation in controlled trials. These preliminary findings support further investigation in controlled trials to determine effectiveness and to formally test hypothesized mechanisms.

## 1. Introduction

The world population is constantly aging. Approximately 25% of the Italian population is over 65 years old, and the median age has increased from about 40 to about 47 years over the past twenty years [1]. Healthy aging and longevity are among the central themes of policy and research in the health and social fields [2]. During old age, in addition to neurocognitive disorders and loss of functional autonomy, psychological distress such as mood disorders, anxiety, adjustment disorders, and somatic symptoms and related disorders are frequent [3,4,5,6,7,8,9,10]. Approximately 10% of older adults have a diagnosis of anxiety or depressive disorder, and about 40% report anxious and/or depressive symptoms [10,11]. The severity of depression is associated with an increased risk of death and suicidal behaviors [12,13]. Emotions and psychological symptoms play a fundamental role in their interaction with the state of physical illness and cognitive impairment [8,14]. Furthermore, the promotion of psychological well-being and quality of life is very important among healthy older adults. Moreover, social isolation and loneliness represent significant risk factors for mental health in older adults, being associated with increased risk of anxiety, depression, cognitive decline, and mortality [2,15]. Given the high prevalence of loneliness in this population (20–40%) [16,17,18,19,20], interventions that combine physical activity with social engagement may be particularly relevant.

Alexithymia is a multidimensional and transdiagnostic construct regarding emotion regulation, characterized by difficulty in identifying and describing one’s own emotions and feelings, and associated with an externally oriented thinking style [21]. The level of alexithymia is higher in older adults than in other age groups [22]. It is strictly related to physical health and somatic symptoms and related disorders [23], as well as to depressive and anxiety symptoms and disorders [24]. Studies have demonstrated that alexithymia is associated with somatization independently of somatic diseases, depression, anxiety, and confounding sociodemographic variables [25]. Alexithymia also seems to depend on low interoceptive accuracy [26].

There is evidence of efficacy for various types of psychological/psychotherapeutic interventions for different clinical conditions and for the promotion of psychological well-being in older adults [27,28,29,30]. However, they require significant resources and expertise, which may present access barriers for older adults [31]. Integrated mind–body interventions could therefore offer a more holistic and sustainable approach to promoting mental health in this population.

The regular practice of moderately intense physical activity is recommended by the World Health Organization to enhance balance, prevent falls, strengthen muscles, and promote psychophysical well-being in adults living independently and with various medical conditions [32]. Studies in older adults have documented that regular physical exercise brings benefits on the physical level and on the cognitive and emotional levels [33,34,35]. Walking programs have shown efficacy in reducing depressive and anxious symptoms, improving cognitive functions, and preventing functional decline [36,37,38].

Mindfulness is defined as the ability to intentionally bring attention to the present moment with a non-judgmental attitude [39]. Mindfulness has shown increasing effectiveness in improving physical and emotional well-being and cognitive functioning in older adults [40,41,42]. In the specific context of somatic symptom disorders, mindfulness and breathing exercises have shown efficacy in improving body awareness and reducing anxiety, depressive symptoms, and pain [43]. Mind wandering, the spontaneous drift of thoughts away from the present moment, can be considered the opposite pole of mindfulness on the same attentional continuum, and is associated with increased rumination, stress, and depressive symptoms [44,45]. In older adults, excessive mind wandering may exacerbate difficulties in emotional regulation and heighten sensitivity to bodily sensations, potentially reinforcing somatic concerns [46]. A meta-analysis of randomized controlled trials exhibited a significant effect of mindfulness-based interventions on alexithymia, with enhanced awareness of bodily sensations identified as a candidate mechanism [47]. Recent studies have further shown that reductions in alexithymia mediate the effects of mindfulness on psychosomatic symptoms [48], suggesting that mindful walking, by fostering present-moment attention to bodily sensations and breathing, may operate through similar pathways [49]. Recent studies on patients with somatic symptom disorder have shown that Mindfulness-Based Cognitive Therapy significantly reduces psychosomatic distress, and that this reduction is mediated by both self-compassion and a decrease in alexithymia, with alexithymia representing a deficit in emotional clarity that interferes with health perception and emotional regulation [48].

Mindful walking integrates the benefits of physical activity and contemplative practice, which consists of walking while intentionally bringing attention to the present moment, to the bodily sensations of movement, and to breathing [39,50]. This practice represents an accessible form of moving meditation, particularly suitable for older adults who may find it difficult to maintain static sitting meditation postures. Studies in adults have documented that mindful walking produces significant improvements in anxiety, distress, depressive symptoms, and psychological quality of life [51,52]. In older adults, however, research remains limited. Available studies have shown promising results on perceived stress and depressive symptoms [53,54], negative affects [55], and some cognitive functions [56], though several reported non-significant findings for depressive symptoms and quality of life [51,54], and most either lacked follow-up assessments [53,56] or showed diminished benefits at follow-up [54]. To date, no study has specifically examined the effects of mindful walking on somatic symptoms or alexithymia in community-dwelling older adults, nor has the sustainability of observed changes been adequately addressed.

The primary aim of this pilot study is to evaluate the feasibility and acceptability of a mindful walking intervention in older adults, including recruitment and retention rates, adherence to the intervention, and participant-reported acceptability [57,58,59,60,61]. The secondary aim is exploratory and is intended to estimate within-person changes in depressive, anxious, and somatic symptoms, as well as in mindfulness-related processes (mindfulness, mind wandering, and alexithymia), in order to inform the design and sample size planning of a future controlled trial. Given the limited availability of longitudinal data in this field, an additional exploratory aim is to examine whether observed within-person changes are maintained at one-month follow-up. Given the pilot and exploratory nature of the study, no confirmatory hypotheses regarding intervention effectiveness are tested. Instead, the following exploratory expectations are examined:

E1. The mindful walking intervention will be feasible and acceptable, as indicated by adequate recruitment and retention rates, high adherence to the intervention sessions, and favorable participant-reported acceptability.

E2. Exploratory within-person reductions in depressive, anxious, and somatic symptoms from pre- to post-intervention will be observed, with corresponding estimates of uncertainty intended to inform future study planning. Moreover, within-person changes in mindfulness, mind wandering, and alexithymia will be examined as candidate processes potentially associated with symptom change.

E3. Changes are expected to be more pronounced in variables theoretically targeted by the intervention, while measures not directly related to mindful walking (e.g., physical, social, and environmental quality of life; relational styles) are expected to show relative stability over time. These patterns are examined descriptively and are not intended to support causal or selective efficacy claims.

## 2. Materials and Methods

### 2.1. Transparency and Openness

We report how we determined our sample size, and describe all data exclusions, manipulations, and all measures in the study, and we follow the Journal Article Reporting Standards (JARS) [62]. All data, analysis code, and research materials are available. Data were analyzed with the PyMC library in Python using Google Colab [63].

### 2.2. Study Design

The study employed a longitudinal repeated-measures pilot design with three assessment points: pre-intervention (A1), post-intervention (A2), and one-month follow-up (A3). All participants took part in the mindful walking program, which consisted of 16 sessions delivered over eight weeks. Psychological measures were collected at each time point to examine within-person changes over time and the short-term maintenance of observed patterns. In line with recommendations for pilot and feasibility studies, the primary purpose of this design was not to test intervention efficacy, but rather to evaluate the feasibility, acceptability, adherence, and safety of the mindful walking program in older adults. Secondary, exploratory outcomes were included to estimate within-person changes in depressive, anxious, and somatic symptoms, as well as in mindfulness-related processes (mindfulness, mind wandering, and alexithymia), with the aim of informing the design, analytic strategy, and sample size planning of a future controlled trial. So, the primary outcomes were feasibility and acceptability indicators, including recruitment, retention, adherence, participant-reported acceptability, and safety/tolerability. Secondary outcomes were exploratory psychological and quality-of-life measures, assessed to estimate within-person changes and generate planning parameters for future controlled research. Given the absence of a control or comparison condition, the study is best classified as a quasi-experimental longitudinal pilot study, and findings related to clinical outcomes are interpreted as exploratory and descriptive rather than confirmatory or causal.

### 2.3. Pilot Study Objectives and Progression Criteria

Consistent with established guidelines for pilot and feasibility research, the study incorporated a predefined pilot framework to evaluate the practicality of the intervention and to inform decisions regarding progression to a subsequent controlled trial.

- Feasibility and acceptability objectives

Predefined feasibility objectives included: (a) recruitment rate, defined as the proportion of eligible participants enrolled relative to those approached; (b) retention, assessed at post-intervention and at one-month follow-up; (c) adherence, operationalized as the number of sessions attended out of the 16 planned sessions; (d) acceptability and perceived burden, assessed through participant-reported satisfaction and qualitative feedback; (e) safety and tolerability, monitored through the recording of adverse events, and physiological responses during the sessions.

- Progression criteria

Progression criteria (“go/no-go”) for a future controlled trial were defined a priori as follows: (a) retention of at least 70–80% of participants at post-intervention; (b) completion of at least 75% of intervention sessions on average; (c) mean acceptability ratings of 4 or higher on a 5-point scale; (d) absence of serious adverse events related to the intervention. In addition, exploratory estimates of within-person change, and their associated uncertainty were examined to evaluate whether the observed signal strength and variability were sufficient to warrant further investigation in a larger, controlled study. Finally, to support the planning of subsequent research, the present pilot study was used to derive descriptive planning parameters, including estimates of outcome variability, pre-post correlations, and participant dropout rates. These parameters are intended to inform methodological considerations (e.g., outcome selection and precision targets) in future controlled research and are not intended as definitive sample size recommendations.

### 2.4. Intervention

After first contact with participants involved in the training, they underwent a screening to select individuals based on their availability to participate in the study, the absence of significant sensory or motor impairments, and the absence of significant limitations in personal/instrumental activities of daily living. This was followed by a pre-test/baseline assessment (Phase A1) evaluating cognitive function, anxiety and geriatric depression symptomatology, quality of life, mindfulness and mind wandering, alexithymia, somatic symptoms, and relational style. Subsequently, participants engaged in sixteen training sessions conducted twice weekly over eight weeks (Phases B1), during which they were involved in a mindful walking practice that lasted approximately 30 min per session. Then, a post-training assessment (Phase A2) and a one-month follow-up assessment (Phase A3) were completed.

In synthesis, the timeline of the training was the following (each test mentioned will be explained in detail later):

(1) Brief training of research assistants on task administration, without disclosing research objectives or expected results.
(2) The screening phase, including screening tool of the Italian version of Integrated Care for Older People (Table 1 of I-COPE) [64,65], motivation and interest questions, Activities of Daily Living (ADL) [66] and Instrumental Activities of Daily Living (IADL) [67].
(3) A1 phase including Montreal Cognitive Assessment (MoCA) [68,69,70,71], Brief Quality of Life Questionnaire (WHO QoL BREF) [72,73], Geriatric Depression Scale (GDS-15) [74,75], State-Trait Anxiety Inventory (STAI-Y) [71], Five Facet Mindfulness Questionnaire (FFMQ) [76], Four Factors of Mind Wandering Questionnaire (4FMWQ) [45], Relationship Questionnaire (RQ) [77], Toronto Alexithymia Scale (TAS-20) [78,79,80,81], and Level 2—Somatic Symptom—Adult Patient (adapted from the Patient Health Questionnaire Physical Symptoms (PHQ-15) [82].
(4) B1 phase: The mindful walking training. Participants also wore a wrist-worn device (Huawei Band 10, Shenzhen, Guangdong, China).
(5) A2 phase: At the end of the training. The same as A1.
(6) A3 phase: One month after the end of the training. The same as A1.

The whole procedure took approximately 16–17 weeks. Details about the procedure are reported in Supplementary Material S1.

### 2.5. Feasibility Assessment

To evaluate the feasibility and tolerability of the mindful walking intervention, two key domains were monitored: physiological adaptation to physical activity and participant-reported acceptability.

Physiological adaptation was assessed through measurements of heart rate (HR) and peripheral oxygen saturation (SpO_2_), obtained prior to each training session. These physiological parameters were monitored to provide descriptive information regarding participants’ cardiovascular responses and overall tolerability to the intervention. Heart rate is widely used as an indicator of cardiovascular demand and exercise intensity, whereas peripheral oxygen saturation provides information about the maintenance of adequate oxygenation during physical activity. The inclusion of these measures was informed by exercise physiology research examining cardiovascular responses and recovery following exercise training [83,84,85]. Given the advanced age of the sample, monitoring HR and SpO_2_ throughout the intervention provided additional information regarding participant safety and physiological adaptation to the mindful walking program. In line with the exploratory nature of this pilot study, these measures were interpreted descriptively and were not intended to provide diagnostic or clinical assessments of cardiopulmonary function.

Acceptability and perceived burden were assessed using a brief, four-item questionnaire developed for this study to capture key dimensions of participant experience. Items were rated on a 5-point Likert-like scale (1 = Strongly Disagree to 5 = Strongly Agree) and addressed the following domains: (a) the overall satisfaction with the mindful walking training; (b) the appropriateness of session duration; (c) structure the perceived impact of the training on personal wellbeing and mindfulness awareness; and (d) the quality of support and guidance provided by the research assistant during sessions. This questionnaire was informed by established client satisfaction frameworks [86,87] and adapted to the context of a home-based mindful walking program. The four-item scale showed satisfactory internal consistency in the present sample (Cronbach’s α = 0.93).

### 2.6. Statistical Analysis

Given the pilot nature of the study and the small sample size, no a priori power calculation was performed. Instead, recruitment was guided by practical considerations, including available resources, participant availability, and the exploratory purpose of the study. Consistent with recommendations for pilot and feasibility research, the primary aim was to assess feasibility, acceptability, and adherence, and to obtain preliminary estimates of outcome variability and within-person change that could inform the design of a future controlled trial, rather than to provide adequately powered tests of intervention effectiveness. The final sample size is consistent with ranges commonly reported in pilot studies and with methodological guidance emphasizing the role of pilot investigations in generating planning parameters for subsequent confirmatory research [59]. Analyses were conducted within a Bayesian Hierarchical Modeling (BHM) to estimate within-person change and associated uncertainty across assessment points (three paired comparisons: Post vs. Pre, Follow-up (FU) vs. Pre, and FU vs. Post), to maximize the informational value of the data and derive planning parameters for future controlled trials. For each outcome and paired comparison, individual difference scores were computed and modeled as arising from Student’s t-distribution, with the mean difference representing the estimated within-person change. This distribution was chosen to provide robustness to potential outliers and to accommodate the limited sample size. Weakly informative priors were used: a Normal (0, 10) prior for the mean difference, a Half-Normal (10) prior for the standard deviation, and an Exponential prior shifted by 1 for the degrees-of-freedom parameter of Student’s t-distribution. These priors were selected to be broad enough to allow the data to drive the posterior estimates while providing regularization appropriate for a small-sample pilot study. Full details of the statistical models and inferential procedures are provided in Supplementary Materials S1. Posterior probabilities were interpreted primarily as continuous measures of evidence regarding the direction of within-person change. For descriptive purposes only, and to facilitate narrative interpretation of the results, the following qualitative labels were adopted: posterior probabilities between 0.75 and 0.89 were described as “possible trends”, values between 0.90 and 0.97 as “probable trends”, and values greater than 0.97 as “likely trends”. These descriptors were used solely as reporting conventions in an exploratory context and do not represent inferential decision thresholds, evidence categories, or criteria for establishing intervention effects [88].

## 3. Results

In line with the pilot nature of the study, results are presented by distinguishing primary feasibility and acceptability outcomes from secondary exploratory estimates of within-person change in psychological and quality-of-life measures.

### 3.1. Sample Characteristics

The sample consisted of 13 older adults, including 7 males and 6 females, with a mean age of 75.40 ± 7.68 years and an average educational level of 8.38 ± 4.81 years. Functional autonomy was largely preserved, as indicated by high scores in both ADL (5.92 ± 0.27) and IADL (7.61 ± 0.65). The mean MoCA score was 21.70 ± 3.47, consistent with preserved global cognitive functioning for the sample. Participants had a mean height of 1.65 m (SD = 0.08), a mean weight of 72.77 kg (SD = 10.43), and a mean Body Mass Index (BMI) of 26.75 kg/m^2^, indicating that, on average, participants were slightly overweight according to standard BMI classifications [89]. Given this characteristic, the mindful walking intervention may have been particularly appropriate, as low-intensity walking is considered a safe and accessible form of physical activity for older adults with mild overweight and has been associated with improvements in metabolic health, mobility, and weight management [90]. None of the participants reported previous formal experience with mindfulness-based interventions, meditation programs, or regular mindfulness practice. Descriptive statistics and results are reported in Table 1.

**Table 1:** Summary of feasibility outcome, sample characteristics, and acceptability measures.

*Feasibility outcomes*	
Enrolled, *n*	15
Pre-intervention withdrawals, *n*	2
Initiated intervention, *n*	13
Adherence	13/13 completed 16/16 sessions (100%)
Retention (post-intervention)	13/13 (100%)
Retention (follow-up)	13/13 (100%)
Serious adverse events	0
Variable	Participants
	(*N* = 13)
*Demographic and clinical characteristics*	
Male/female	7/6
Age, years	75.40 ± 7.68
Education, years	8.38 ± 4.81
Activities of daily living	5.92 ± 0.27
Instrumental activities of daily living	7.61 ± 0.65
Montreal Cognitive Assessment	21.70 ± 3.47
*Physiological and acceptability measures*	
HR (pre)	69.17 ± 9.01
HR (post)	74.48 ± 10.44
SpO_2_ (pre)	94.62 ± 6.50
SpO_2_ (post)	96.09 ± 2.63
Overall satisfaction	4.14 ± 0.74
Session structure and duration	4.14 ± 0.76
Perceived effectiveness	4.39 ± 0.67
Support from research assistant	4.36 ± 0.67

### 3.2. Feasibility and Acceptability Outcomes

Feasibility was evaluated across predefined domains, including recruitment, retention, adherence, acceptability, and safety/tolerability.

- Recruitment

Fifteen eligible older adults consented to participate and were enrolled in the study. Of these, 13 participants initiated the mindful walking intervention and were included in the analyses. Two participants withdrew prior to intervention initiation and were therefore not considered dropouts.

- Retention

All participants who initiated the intervention completed the post-intervention assessment (retention rate = 100%). At the one-month follow-up, 13 out of 13 participants completed the assessment, corresponding to a retention rate of 100%.

- Adherence

Adherence to the intervention protocol was complete. All 13 participants who initiated the intervention attended all 16 planned sessions, corresponding to an adherence rate of 100%. No participant discontinued the intervention after initiation.

- Acceptability

Participant-reported acceptability was high across all questionnaire items. Mean scores ranged from 4.14 to 4.39 on a 5-point Likert scale, with standard deviations between 0.67 and 0.76, indicating consistently positive evaluations. The highest ratings were observed for perceived effectiveness of the training and the quality of support provided during sessions, followed by overall satisfaction with the intervention and perceived appropriateness of session structure and duration.

- Safety and tolerability

No serious adverse events related to the intervention were reported. Physiological tolerability was further supported by the stability of cardiovascular and oxygenation parameters throughout the intervention period. Mean resting heart rate increased modestly from approximately 69 beats/min before the intervention to 74 beats/min at post-intervention, while peripheral oxygen saturation remained high and stable, increasing approximately from 95% to 97%. These findings suggest that participants tolerated the mindful walking program without evidence of clinically relevant physiological deterioration. Given the advanced age of the sample, the maintenance of adequate oxygenation levels and stable cardiovascular responses provides additional support for the feasibility and safety of the intervention [85,91,92].

Together, these findings indicate that the mindful walking intervention was feasible, well-tolerated, and acceptable in this sample of older adults, meeting the predefined primary objectives of this pilot study.

### 3.3. Exploratory Within-Person Changes

All secondary outcomes are reported as exploratory estimates of within-person change derived from the Bayesian hierarchical models. Given the pilot design and the absence of a control condition, results are presented descriptively and are not intended to support confirmatory or causal conclusions. Results are reported in Table 2.

#### 3.3.1. Depressive Symptoms

Exploratory analyses of the GDS suggested a likely reduction in depressive symptoms following the intervention. This pattern was evident both immediately after the intervention and at one-month follow-up, indicating that the observed improvement was maintained over time. Comparisons between post-intervention and follow-up scores suggested relative stability after intervention completion (Table 2).

#### 3.3.2. State Anxiety and Trait Anxiety

STAI-S analyses suggested no meaningful changes in state anxiety across the study period. In contrast, trait anxiety showed a likely reduction immediately following the intervention; however, this pattern was not maintained at follow-up, with scores returning toward baseline levels. Overall, the findings suggest a transient improvement in trait anxiety but no consistent change in state anxiety (Table 2).

#### 3.3.3. Somatic Symptoms

Exploratory analyses of PHQ-15 suggested a tendency toward reduced somatic symptom reporting following the intervention. Improvements were observed both immediately after the intervention and at follow-up, with the most consistent pattern emerging at follow-up relative to baseline. Somatic symptom levels remained relatively stable between post-intervention and follow-up, suggesting maintenance of the observed improvement over time (Table 2).

#### 3.3.4. Mindfulness and Mind Wandering

Exploratory analyses of the FFMQ suggested a substantial increase in mindfulness following the intervention, which was maintained at follow-up. Mind wandering showed a directional tendency toward reduction at both post-intervention and follow-up assessments; however, uncertainty regarding the magnitude of this change remained considerable. Overall, the findings suggest sustained improvements in mindfulness accompanied by a possible reduction in mind wandering over time (Table 2).

#### 3.3.5. Alexithymia

Exploratory analyses suggested a likely reduction in alexithymia following the intervention. This pattern was observed at both post-intervention and follow-up assessments, indicating maintenance of the observed improvement over time (Table 2).

#### 3.3.6. Quality of Life

Exploratory analyses of quality-of-life outcomes revealed a differentiated pattern across domains. The Psychological QOL dimension showed the most consistent positive pattern, with likely improvements observed following the intervention and maintained at follow-up. In contrast, the Physical, Social, and Environmental QOL dimensions showed only small-to-moderate positive tendencies characterized by substantial uncertainty. Across these domains, patterns generally suggested stability or maintenance of any observed changes between post-intervention and follow-up assessments. Overall, the findings indicate that improvements were more pronounced for psychological quality of life than for physical, social, or environmental quality-of-life domains (Table 2).

### 3.4. Relationship Questionnaire

Exploratory Bayesian analyses of attachment styles did not indicate systematic within-person changes in attachment styles across the intervention or follow-up period. Across all four Relationship Questionnaire styles (A, B, C, and D), posterior estimates suggested substantial uncertainty and no consistent evidence of meaningful change. Although some comparisons showed directional tendencies, these patterns were not sufficiently robust to support clear interpretation and should be considered exploratory (Table 2).

## 4. Discussion

This study represents a pilot feasibility evaluation of a mindful walking intervention in community-dwelling older adults, aimed at assessing acceptability, tolerability, and feasibility, and at generating exploratory estimates of within-person change across psychological and quality-of-life outcomes. In line with the pilot nature of the study and the absence of a control condition, results should be interpreted as preliminary and descriptive, intended to inform hypotheses and the design of future controlled trials rather than to establish intervention efficacy.

Overall, findings indicate that the intervention was highly feasible and well accepted and revealed consistent exploratory patterns of change in several clinically relevant domains, including depressive symptoms, psychological quality of life, somatic symptoms, alexithymia, and mindfulness, some of which were maintained at one-month follow-up. Feasibility was supported across all predefined domains. Recruitment targets were met, and retention was optimal across both assessment points, exceeding commonly recommended thresholds for pilot studies and suggesting that the assessment schedule and study burden were well tolerated in this population. Adherence to the intervention protocol was exceptionally high; while this may partly reflect the small sample size and the supportive role of the research assistant, it nonetheless demonstrates that session frequency, duration, and structure were manageable and acceptable for older adults, and suggests that these features do not represent major barriers to participation or scalability. Acceptability ratings were consistently positive across all domains and above the predefined progression threshold; the pattern of responses aligns with established client satisfaction frameworks and supports the perceived relevance and usability of the mindful walking approach in this age group. No serious adverse events were reported, and the physiological indexes remained within expected ranges throughout the intervention, suggesting adequate physiological adaptation rather than excessive strain, findings particularly relevant given the older age of the sample and informative for the design of safety monitoring procedures in future trials. Taken together, all predefined progression (“go/no-go”) criteria were met, supporting the justification for advancing to a larger, controlled study.

Our findings regarding depressive symptoms are broadly consistent with previous literature documenting associations between walking interventions and reductions in depressive symptoms in adults [93], as well as with studies suggesting benefits of mindfulness-based approaches in older populations [42]. Within this context, the present pilot study extends existing work in several exploratory directions. First, the observed within-person patterns suggest that the integration of mindfulness with walking may be associated with maintained reductions in depressive symptoms, addressing some of the heterogeneity reported in previous studies that yielded non-significant or mixed results [51,54]. Second, the Bayesian analytic framework allowed for the estimation of the magnitude, direction, and uncertainty of change, providing probabilistic information that is particularly informative in small-sample pilot research. Third, unlike prior studies that focused on a limited number of outcomes [51,53,54,55,56], the present study employed a broader assessment battery, revealing a pattern of selective rather than generalized within-person change across psychological and quality-of-life domains.

Improvements in psychological quality of life mirrored and complemented the observed reductions in depressive symptoms, suggesting that the intervention was associated not only with changes in symptom reporting but also with enhancements in positive aspects of psychological functioning. This pattern is particularly relevant in older adulthood, where the promotion of well-being represents an important complement to symptom management [29]. In the context of a pilot design, the observed posterior patterns—characterized by consistent directionality and maintenance at follow-up—suggest that psychological quality of life may represent a sensitive outcome domain for future controlled investigations of mindful walking.

A complex and theoretically interesting pattern emerged for anxiety. A marked reduction in trait anxiety was observed immediately post-intervention; however, these findings should be considered exploratory, but this pattern was not maintained at one-month follow-up, with levels returning substantially to baseline. State anxiety, conversely, showed no meaningful changes at any time point. This dissociation is consistent with previous longitudinal research demonstrating that meditation practice produces significant reductions in trait anxiety, but not state anxiety, when measured at intervals of several weeks [93,94]. The distinction between these constructs is relevant for interpretation: state anxiety is a transitory emotional response to specific circumstances, while trait anxiety refers to a stable dispositional tendency to perceive stressors as threatening [71,95], and has been identified as a key susceptibility phenotype influencing both mental health and intervention responses [94]. The absence of change in state anxiety may reflect the fact that point-in-time assessments conducted during formal sessions did not capture fluctuations during daily life or actual practice. Conversely, the trait anxiety measure, requiring a retrospective aggregated evaluation, may have been more sensitive to the accumulation of positive experiences over eight weeks, consistent with evidence that increases in mindfulness preceded and mediate reductions in trait anxiety through mechanisms such as decentering and cognitive defusion [93,94]. The loss of benefit at follow-up suggests that the observed change required continued practice to be sustained, consistent with theoretical models emphasizing practice dependency for maintaining mindfulness benefits [39,93]. More cautious interpretations must also be considered: the post-intervention reduction could partly reflect response biases, and the absence of a control group does not allow us to rule out contributions from nonspecific factors such as attention, expectations, or regression to the mean. In summary, the pattern suggests that mindful walking may have influenced trait anxiety through enhanced mindfulness and metacognitive awareness [93,94], though this benefit was not reflected in state anxiety and was not maintained without continued practice, underscoring the importance of strategies to support autonomous practice after program completion, such as booster sessions or community-based practice groups. However, several alternative explanations for this pattern must also be acknowledged. The post-intervention reduction in trait anxiety may partly reflect a transient, context-specific response tied to the structured group environment and the regularity of sessions, rather than a durable dispositional change. Similarly, the social support inherent in the group format may have contributed to a temporary sense of containment that diminished once the program concluded. A measurement artifact cannot be fully excluded either, as the retrospective format of the STAI-T may be sensitive to recency effects, potentially amplifying the perceived benefit immediately post-intervention. Critically, the absence of systematic monitoring of between-session practice represents a key interpretive limitation for this finding: it is not possible to determine whether the reversal observed at follow-up reflects discontinued or reduced autonomous practice, or whether it is attributable to other processes unrelated to the intervention.

One of the most theoretically informative patterns observed in this pilot study concerns the concomitant and sustained reduction in somatic symptoms and alexithymia. Alexithymia, defined as difficulty in identifying and describing one’s emotions and bodily sensations, represents a candidate process that may be involved in the pathway linking mindful walking to somatic symptom change; however, given the absence of formal mediation analysis, this possibility should be interpreted strictly as a hypothesis for future testing rather than an inferred mechanism. Several convergent observations support this hypothesis. First, both variables showed consistent within-person reductions at post-intervention and follow-up, though their co-occurrence does not establish mediation. Second, mindful walking, through focused attention to bodily sensations during movement and breathing, may have enhanced interoceptive awareness, which could in turn facilitate the recognition and description of emotional states characteristic of lower alexithymia, consistent with meta-analytic evidence identifying bodily awareness as a candidate mechanism of mindfulness-based interventions on alexithymia [47]. Third, recent studies have demonstrated that reductions in alexithymia statistically mediate the effects of mindfulness on psychosomatic symptoms in patients with somatic symptom disorder [48], providing theoretical grounds for a similar hypothesis in community-dwelling older adults. Formal mediation models using pre-registered designs and adequate sample sizes will be needed to test this rigorously in future controlled trials. Finally, the concomitant reduction in alexithymia and depressive symptoms is consistent with the well-established bidirectional association between these constructs, whereby changes in each may partially depend on and reciprocally influence the other [96], a relationship that should be kept in mind when interpreting co-occurring within-person changes.

Further consideration concerns the interpretation of the outcomes showing the strongest within-person changes, particularly depressive symptoms (GDS) and alexithymia (TAS-20). Although Bayesian analyses yielded high posterior probabilities for reductions in these measures, such probabilities reflect the degree of certainty regarding observed within-person change and should not be interpreted as evidence of intervention efficacy. In the absence of a control group, several alternative explanations remain plausible, including Hawthorne effects, expectancy effects, regression to the mean, maturation processes, repeated assessment effects, social engagement associated with study participation, and natural symptom fluctuations over time. For depressive symptoms, participation in a structured and socially meaningful activity, together with positive expectations regarding the intervention, may have contributed to improved self-reported mood independently of any specific effect of mindful walking. Similarly, reductions in alexithymia may have been influenced by repeated exposure to self-report measures focusing on emotions and internal experiences, increased self-reflection during study participation, or by concurrent changes in depressive symptom reporting. Consequently, the observed changes cannot be unequivocally attributed to the intervention itself. The selective pattern of change across outcomes should therefore be interpreted as a descriptive observation rather than evidence against these alternative explanations. Accordingly, all findings should be considered exploratory estimates of within-person change that may inform future hypothesis generation and the design of randomized controlled trials with appropriate comparison conditions.

Consistent with these exploratory observations, a theoretical model proposes that mindful walking might operate through a pathway in which enhanced bodily awareness reduces alexithymia, which in turn may facilitate lower somatic symptom reporting, a model suggested, not confirmed, by the present data, and requiring formal testing in future studies. The within-person increases in dispositional mindfulness observed in this study provide a further exploratory signal, suggesting that the intervention may have supported the cultivation of present-moment attentional capacity, although the absence of a control group precludes causal attribution. Parallel to this, a directional tendency toward reduced mind wandering was observed, supporting the theoretical framework conceptualizing mindfulness and mind wandering as opposite poles of an attentional continuum [46]. These co-occurring patterns are theoretically coherent, as mind wandering is associated with increased rumination, stress, and depressive symptoms [44], while mindfulness promotes enhanced emotional regulation and reduced reactivity to disturbing thoughts and sensations.

Consistent with E3, the pattern of findings suggests selective rather than generalized exploratory change. The physical, social, and environmental dimensions of quality of life showed positive trends with moderate posterior probabilities, but without reaching robust credibility thresholds, suggesting that mindful walking may be associated with more pronounced exploratory changes in psychological and emotional aspects of well-being, while its relationship with more contextual and relational dimensions appears more modest. Similarly, attachment styles showed no credible within-person changes, consistent with the expectation that the intervention would not modify more stable personality characteristics. These null findings are theoretically coherent and contribute to the internal consistency of the results, suggesting that mindful walking may preferentially influence specific psychological processes rather than producing uniform non-specific effects. However, given the study design, alternative explanations cannot be ruled out, and all findings should be interpreted as exploratory.

The findings of the present pilot study have relevant clinical and practical implications for the promotion of psychological well-being in older adults, primarily by demonstrating that mindful walking is a feasible, acceptable, and well-tolerated intervention in this population. In line with this, structured outdoor walking and exercise programs have also been shown to promote improvements in balance and physical performance in older adults [97], further supporting the broader health relevance of walking-based interventions in this population.

Mindful walking appears particularly suitable for older adults for several reasons. First, it is highly accessible: it requires no specialized equipment, can be practiced autonomously at home or in natural settings, and does not impose physical demands that might limit participation in more intensive forms of exercise or static meditation practices. Consistent with this, physiological indicators remained within normal ranges throughout the intervention, suggesting good cardiovascular tolerability. Second, the intervention showed high acceptability, with consistently elevated satisfaction ratings across all domains, indicating that participants perceived the program as useful, engaging, and appropriately structured. Third, the brief format (16 sessions over 8 weeks) and the possibility of delivery by operators with relatively limited training suggest that mindful walking may be scalable and implementable within community mental health services, senior centers, and health promotion programs. Within the limits of a pilot design, the observed exploratory patterns of change in depressive symptoms, somatic complaints, alexithymia, and mindfulness suggest that mindful walking may represent a promising candidate intervention for older adults experiencing subsyndromal psychological distress, as well as a potential preventive approach to support healthy aging. However, these implications should be confirmed in future controlled trials before integration into routine care pathways.

Several methodological limitations must be considered when interpreting the findings of this pilot study. First, the absence of a control group represents a substantial limitation that precludes causal inference. Alternative explanations for the observed within-person changes, including Hawthorne effects, expectancy effects, maturation, regression to the mean, repeated assessment effects, acquiescence bias, and other non-specific influences such as the passage of time and attention received from intervention facilitators, cannot be ruled out. Consequently, all observed changes should be interpreted as exploratory estimates rather than evidence of intervention efficacy. Future studies should include active control conditions (e.g., walking without mindfulness and/or mindfulness without walking) to disentangle the specific contributions of each intervention component. Furthermore, an active control condition matched for mindful walking but without the social component would help clarify the extent to which the observed effects are attributable to mindfulness and walking per se, rather than to the benefits of social interaction inherent in group-based activities. Second, despite the use of Bayesian analytic methods suitable for small samples, the limited sample size (*N* = 13) restricts the generalizability of findings, which may not extend to more frail, institutionalized, or cognitively or physically impaired older adults. Third, the one-month follow-up period may be insufficient to assess the long-term sustainability of observed patterns, as is particularly evident for trait anxiety, where initial benefits were already lost at one month. Fourth, all outcome measures were self-reported, introducing potential biases related to expectations, social desirability, or poor introspection. The inclusion of objective measures would substantially strengthen the validity of future results. Fifth, adherence to autonomous practice between supervised sessions was not systematically monitored, making it impossible to establish a dose–response relationship or to determine whether the reversal of trait anxiety benefits at follow-up reflects discontinued practice or other processes; future studies should incorporate structured practice diaries or ecological momentary assessment to address this limitation. Finally, the simultaneous examination of a large number of exploratory outcomes, in the absence of pre-registration and clearly defined primary versus secondary outcomes, substantially increases the risk of false positive findings; all reported patterns should therefore be interpreted as preliminary hypothesis-generating signals to be formally tested in future pre-registered, adequately powered trials with a limited set of pre-specified outcomes.

Future research should build on these preliminary findings through randomized controlled trials with larger samples, longer follow-up periods, and active control conditions. Such studies should prioritize a limited number of prespecified primary outcomes, formally test candidate mechanisms using appropriate mediation models, integrate objective outcome measures, and evaluate strategies to support the maintenance of benefits after program completion, such as booster sessions or community-based practice formats. Testing the intervention in more heterogeneous and clinically complex older populations will also be essential to assess its broader applicability.

## 5. Conclusions

In conclusion, this study provides a pilot feasibility evaluation of a mindful walking intervention in community-dwelling older adults, demonstrating high acceptability, tolerability, and adherence, together with exploratory within-person patterns suggesting possible improvements in depressive symptoms, somatic complaints, alexithymia, and mindfulness. Some of these patterns were maintained at one-month follow-up. Within the constraints of a single-arm pilot design, these preliminary and descriptive signals do not constitute evidence of efficacy and require confirmation through adequately powered randomized controlled trials before any clinical conclusions can be drawn. By integrating an accessible form of physical activity with contemplative practice, mindful walking represents a promising candidate intervention worthy of further investigation for promoting psychological well-being in older adulthood. In addition, this work contributes to ongoing efforts within geropsychology to conceptualize the psychological needs of older adults from a perspective that is not merely an extension of clinical psychology models developed for younger populations [98]. Although methodological limitations, particularly the absence of a control group and the small sample size, preclude causal conclusions, the observed pattern of selective exploratory changes, together with the strong feasibility data, supports the continuation of research on mindful walking using more rigorous controlled designs. With appropriate methodological refinements, future studies will be able to determine whether mindful walking can be effectively integrated into community mental health and health-promotion services for older adults.

## Figures and Tables

**Table 2:** Bayesian analysis of outcome comparisons.

	Comparison	Mean Difference (Δ)	SD	94% HDI	Posterior Probability (Δ > 0)	Interpretation
QOL_Physical	Post vs. Pre	0.75	0.73	[−0.60, 2.12]	0.854	Directional positive pattern with substantial uncertainty
	FU vs. Pre	0.81	0.66	[−0.44, 2.06]	0.906	Positive tendency with considerable uncertainty
	FU vs. Post	0.18	0.78	[−1.32, 1.63]	0.596	No meaningful change
QOL_Social	Post vs. Pre	0.36	0.50	[−0.80, 1.41]	0.749	No meaningful change
	FU vs. Pre	0.61	0.60	[−0.52, 1.74]	0.861	Directional positive pattern with substantial uncertainty
	FU vs. Post	0.29	0.66	[−0.97, 1.53]	0.68	No meaningful change
QOL_Environment	Post vs. Pre	0.53	0.62	[−0.64, 1.71]	0.816	Directional positive pattern with substantial uncertainty
	FU vs. Pre	0.87	0.59	[−0.24, 1.98]	0.934	Positive tendency with considerable uncertainty
	FU vs. Post	0.48	0.62	[−0.97, 1.53]	0.785	No meaningful change
QOL_Psychological	Post vs. Pre	2.88	0.53	[1.81, 3.81]	1	Likely positive trend
	FU vs. Pre	2.04	0.78	[0.64, 3.60]	0.992	Likely positive trend (maintained at follow-up)
	FU vs. Post	0.00	0.00	[0.0, 0.0]	0.491	No change (scores remained stable)
Geriatric Depression Scale	Post vs. Pre	−1.89	0.52	[−2.87, −0.90]	0.999	Likely negative trend (lower depression)
	FU vs. Pre	−2.01	0.65	[−3.19, −0.75]	0.996	Sustained improvement
	FU vs. Post	−0.16	0.30	[−0.74, 0.42]	0.705	No meaningful change (improvement maintained)
State Anxiety Inventory	Post vs. Pre	0.60	1.08	[−1.34, 2.73]	0.279	No meaningful change
	FU vs. Pre	0.38	1.03	[−1.59, 2.34]	0.352	No meaningful change
	FU vs. Post	−0.24	0.75	[−1.68, 1.16]	0.635	No meaningful change
Trait Anxiety Inventory	Post vs. Pre	−4.43	0.82	[−6.03, −3.00]	1	Likely negative trend (reduced trait anxiety)
	FU vs. Pre	0.52	1.99	[−3.27, 4.19]	0.386	No meaningful change
	FU vs. Post	5.00	2.14	[0.90, 8.94]	0.010	Clear worsening (trait anxiety increased)
Five Facet Mindfulness Questionnaire	Post vs. Pre	9.12	2.052	[5.87, 13.61]	1	Likely positive trend
	FU vs. Pre	9.12	2.052	[5.87, 13.61]	1	Likely positive trend (fully maintained)
	FU vs. Post	0.00	0.00	[0.0, 0.0]	0.506	No change (stable over time)
Four Factors of Mind Wandering Questionnaire	Post vs. Pre	−2.36	1.83	[−5.86, 1.12]	0.907	Directional tendency toward reduction with substantial uncertainty (reduction in somatic symptoms)
	FU vs. Pre	−2.73	1.73	[−5.95, 0.51]	0.948	Directional tendency toward reduction with substantial uncertainty (reduction in somatic symptoms)
	FU vs. Post	−0.66	1.04	[−2.71, 1.27]	0.754	No meaningful change
Relationship Questionnaire Style A	Post vs. Pre	0.64	0.96	[−1.17, 2.51]	0.754	No meaningful change
	FU vs. Pre	0.55	0.92	[−1.18, 2.31]	0.735	No meaningful change
	FU vs. Post	−0.02	0.80	[−1.53, 1.55]	0.478	No meaningful change
Relationship Questionnaire Style B	Post vs. Pre	1.12	0.649	[−0.11, 2.35]	0.043	Small directional tendency with low precision
	FU vs. Pre	0.89	0.90	[−0.82, 2.61]	0.151	No meaningful change
	FU vs. Post	−0.29	0.53	[−1.33, 0.72]	0.716	No meaningful change
Relationship Questionnaire Style C	Post vs. Pre	−0.79	0.43	[−1.61, 0.02]	0.967	Uncertain tendency toward reduction in C-style attachment
	FU vs. Pre	−0.84	0.75	[−2.30, 0.59]	0.884	No meaningful change
	FU vs. Post	−0.04	0.52	[−1.02, 0.95]	0.538	No meaningful change
Relationship Questionnaire Style D	Post vs. Pre	1.23	0.97	[−0.57, 3.14]	0.094	No meaningful change
	FU vs. Pre	1.34	0.82	[−0.23, 2.93]	0.05	Borderline positive trend
	FU vs. Post	0.03	0.88	[−1.61, 1.69]	0.487	No meaningful change
Toronto Alexithymia Scale	Post vs. Pre	−5.46	2.61	[−10.28, −0.42]	0.977	Directional tendency toward reduction with substantial uncertainty (reduction in somatic symptoms)
	FU vs. Pre	−7.15	1.83	[−10.31, −3.50]	1	Likely negative trend
	FU vs. Post	−0.86	2.45	[−5.59, 3.56]	0.635	No meaningful change
Somatic Symptom—Adult Patient	Post vs. Pre	−0.95	0.62	[−2.19, 0.15]	0.943	Directional tendency toward reduction with substantial uncertainty (reduction in somatic symptoms)
	FU vs. Pre	−1.47	0.67	[−2.77, −0.24]	0.984	Likely negative trend
	FU vs. Post	−0.47	0.52	[−1.46, 0.51]	0.832	No meaningful change

Note: For outcomes where lower scores indicate improvement (GDS, PHQ-15, TAS-20, 4FMWQ, STAI), posterior probabilities refer to P(Δ < 0). For outcomes where higher scores indicate improvement (FFMQ, QOL), posterior probabilities refer to P(Δ > 0). Posterior probabilities are reported as continuous measures of evidence regarding the direction of change. The qualitative descriptors “possible”, “probable”, and “likely” are descriptive reporting conventions adopted to facilitate interpretation (0.75–0.89, 0.90–0.97, and >0.97, respectively) and do not represent inferential decision thresholds or evidence categories.

## Data Availability

Data can be obtained from the first author by email request.

## Supplementary Materials

The following supporting information can be downloaded at: https://www.mdpi.com/article/10.3390/ijerph23070836/s1. Supplementary Materials S1: Details about the procedure [99,100,101,102,103,104,105,106,107,108].
