# Supplementary material for: A Pilot Feasibility Study of Mindful Walking in Older Adults: Exploratory Bayesian Estimates of Psychological Distress and Alexithymia
Source: Int J Environ Res Public Health. 2026 Jun 25;23(7):836. doi: 10.3390/ijerph23070836 (PMC13410204; doi:10.3390/ijerph23070836)
Supplement: Supplementary file 1 [file ijerph-23-00836-s001.zip › ijerph-4343626-supplementary.pdf]

Supplementary Materials S1

Details about procedure

1. *Screening*. Fifteen individuals were initially contacted in March 2023. All participants were from the city of Bari, Apulia, Italy. Participants were drawn from a pre-existing pool of individuals including people from lifelong-learning universities and from partner associations with which we had established training collaborations, who were interested in taking part in our studies. The proposal received two rejections. All participants, blind to the general hypothesis of the study, signed a written consent form. The Ethical Committee of the Institution approved the study protocol (ET 21-01), and the whole study was performed following the Helsinki Declaration and its later amendments. Then, an initial screening was conducted using the Table 1 of I-COPE tool (for further details: https://www.who.int/publications/i/item/WHO-FWC-ALC-19) excluding people with impairment in four of the six intrinsic capacities measured: vision, hearing, vitality (nutrition), and mobility. Whereas those with cognitive difficulties (mild impairments on memory or time and space orientation) or psychological issues (depression symptoms) were not excluded. All participants were retained for the study. Moreover, they were evaluated for Activities of Daily Living (ADL) and Instrumental Activities of Daily Living (IADL). ADL evaluates six basic self-care tasks (i.e., mean ± sd 5.92 ± 0.27), while IADL assesses more complex, autonomy-related functions like managing finances and medications (i.e., mean ± sd 7.61 ± 0.65). Cut-off scores were >4 for ADL, >4 for men, and >6 for women on the IADL, with high reliability (0.90 and 0.85, respectively). Difficulties in IADLs are often early indicators of mild cognitive impairment and dementia (Pérès et al., 2008; Marshall et al., 2011), while ADL decline signals more advanced stages (Gauthier et al., 2006). While these tools confirm general independence, the study prioritizes sustained moderate physical and cognitive activity as key to preserving overall autonomy. No participant was excluded. The final sample was composed of

thirteen participants (6 women) (i.e., age mean ± sd 75.40± 7.68; level of education mean ± sd 8.38 ± 4.81). Descriptive statistics are reported in Table 1.

2. *A1 baseline assessment.* To evaluate general *cognitive functioning*, MoCA was employed. It is a 30-point screening tool that takes about 10 minutes and evaluates multiple cognitive domains, including memory, visuospatial skills, executive functions, attention, language, and orientation. It has a reliability of 0.83. A cut-off score of 17 was adopted, as proposed by Bosco et al. (2017), with a sensitivity of 0.85, showing the proportion of correctly identified cases with probable cognitive impairment, and a specificity of 0.64, representing the percentage of healthy individuals correctly identified. To evaluate *wellbeing* the thirteen participants were administered the brief quality of life questionnaire WHOQOL-BREF coded into its four domains (physical health, psychological health, social relationships, and environment). This tool allowed for a multidimensional evaluation of the participants' perceived quality of life. Each domain is calculated by averaging the scores of the items within that domain. The mean scores can be easily equated with the WHOQOL-100 scale. The internal consistency of the WHOQOL-BREF, measured through Cronbach's alpha, varies across domains: Physical Health: α = 0.80; Psychological Health: α = 0.75; Environment: α = 0.73; Social Relationships: α = 0.65. The inclusion of the four domains provided a comprehensive understanding of changes in physical, psychological, social, and environmental well-being, while the global items allowed an overarching evaluation of participants perceived quality of life and general health over the course of the intervention. Research indicates that a score below 60 on the WHOQOL-BREF effectively signals poor QoL among older individuals, as demonstrated in various studies (e.g., Silva et al., 2014; Hidayati et al., 2018). In addition, to capture *emotional and psychological dimensions* several questionnaires were used.

   - ✓ The 15-item version of GDS was used, which. The scale measures depressive symptoms and assesses emotional well-being in older adults,

with a cutoff of more than five and a Cronbach's alpha of 0.91. The GDS-15 is also noted for a pooled sensitivity of 0.89 and specificity of 0.77 at this cut-off in Pocklington and colleagues (2016).

- ✓ The STAI-Y, a self-report questionnaire consisting of two 20-item scales providing separate measures of state and trait anxiety (S-Anxiety and T-Anxiety, respectively), was used. S-Anxiety is a transitory response to an event perceived as adverse, characterized by feelings of tension, apprehension, nervousness, and worry. On a 4-point Likert scale (1–4), a score equal to 4 indicates the presence of a higher level of anxiety. The scale showed a good internal consistency of 0.90.
- ✓ The FFMQ consisted of 39 items measuring five facets of mindfulness: (a) describing/labelling with words, the individual's capacity to recognize and label the thoughts and feelings they experience; (b) observing/noticing/attending to sensations/perceptions/thoughts/feelings, the individual's tendency to be aware of and recognize their thoughts and feelings; (c) acting with awareness, the individual's ability to stay present and aware in the moment while ignoring or sidestepping potential distractions; (d) nonjudging of experience, the tendency towards objective consideration of thoughts and feelings and the rejection of assigning value to these thoughts and feelings; and (e) nonreactivity to inner experiences, the individual's ability to remain calm and objective when faced with thoughts or feelings that may usually elicit emotional responses. The FFMQ had high internal consistency with an alpha of .90 (Cronbach's alpha coefficient of each subscale was .93 in observing, .73 in describing, .87 in acting with awareness, .88 in nonjudging of experience and .77 in nonreactivity). The final score was based on a Likert-like scale from 1 (never or very rarely true) to 5 (very often or always true) obtained by summing up responses. Higher scores on the FFMQ indicated, also, a greater tendency to be mindful

- ✓ The 4FMWQ was used to assess unintentional mind wandering. The 16 items cover four factors: (a) Failure in social interaction, (b) Failure in interaction with objects, (c) Unawareness and (d) Inattention. The questionnaire showed a good internal consistency of 0.82.
- ✓ The adult attachment styles were assessed using the RQ. It is a brief, self-report instrument based on the four-category model of adult attachment, which considers two underlying dimensions: the Model of Self (positive or negative) and the Model of Other (positive or negative). The measure consists of four short paragraphs, each describing a prototypical adult attachment pattern: Secure: Characterized by comfort with closeness and interdependence (Positive Self, Positive Other); Preoccupied: Characterized by a strong desire for intimacy coupled with a fear of rejection (Negative Self, Positive Other); Dismissing: Characterized by high self-reliance and a tendency to minimize close relationships (Positive Self, Negative Other); Fearful: Characterized by a desire for close relationships but discomfort with both intimacy and dependence (Negative Self, Negative Other). Participants are asked to rate their degree of correspondence to each of the four prototypes on a 7-point Likert scale (e.g., 1 = *Not at all like me* to 7 = *Very much like me*). These four ratings provide a continuous score for each attachment style. While the measure can be used for categorical classification, it is most employed to obtain continuous scores reflecting the degree to which an individual exhibits characteristics of each of the four styles.
- ✓ The TAS-20 was administered. It is a self-report instrument comprising items assessed on a 5-point Likert scale (ranging from 1 = "strongly disagree" to 5 = "strongly agree"). The 20-item measure encompasses three subscales: difficulty in identifying feelings (DIF); difficulty in describing feelings to others (DDF); and externally oriented thinking (EOT). Total

scores (TAS-20-TS) range from 20 to 100, with higher scores indicating greater alexithymia severity.

- ✓ Somatic symptom severity was assessed using the Level 2- Somatic Symptom-Adult Patient scale, a measure adapted from the Patient Health Questionnaire Physical Symptoms (PHQ-15). The scale is a brief, self-administered instrument used to screen for somatization and monitor the severity of somatic symptoms over the past seven days. The measure consists of 15 items, each corresponding to a common somatic complaint (e.g., stomach pain, trouble sleeping, feeling tired). Respondents rate the extent to which they have been bothered by each symptom on a 3-point Likert scale: 0 = Not bothered at all, 1 = Bothered a little, and 2 = Bothered a lot. Total scores range from 0 to 30, with higher scores indicating greater somatic symptom severity. Consistent with established cut-offs for the PHQ-15, scores were interpreted as follows: minimal (0–4), low (5–9), medium (10–14), and high (15–30) somatic symptom severity.

3. *B1 Phase*. The mindful walking exercise implemented in this study was adapted from previously validated mindfulness-based walking protocols (e.g., Teut et al., 2013; Gotink et al., 2016), which combine meditative awareness with gentle physical activity and sensory grounding. The training aimed to cultivate focused attention on bodily sensations, breathing, and environmental stimuli while walking in everyday settings. During the training phase, participants engaged in a structured 30-minute mindful walking exercise designed to enhance bodily and respiratory awareness. The sessions were conducted by research assistants trained in clinical psychology who had completed a two-week training program prior to leading the exercises. Each session began at home with a brief guided breathing practice, followed by a mindful walk. At home, participants were instructed to sit comfortably with an upright but relaxed posture and focus on their natural breathing. They were guided to observe the movement of the abdomen while inhaling and exhaling, cultivating nonjudgmental awareness of

the breath. Breathing exercises followed a *triangular breathing* pattern: inhaling slowly through the nose while mentally counting to three or four, then exhaling for the same duration. Participants were invited to extend the duration of the breathing cycle as they felt comfortable, maintaining a calm and regular rhythm. The goal of this preparatory exercise was to foster attentional focus, relaxation, and awareness of internal sensations before beginning the walk. Following the breathing practice, participants performed a 30-minute mindful walk starting from their homes. They walked for approximately 15 minutes in one direction and then returned along the same route for another 15 minutes. The route typically included pedestrian streets and green areas such as local parks, allowing participants to practice in safe and calm environments. Participants were instructed to walk at a comfortable, self-selected pace corresponding to light-to-moderate physical exertion, while maintaining the ability to breathe comfortably and sustain attention to bodily sensationsSessions were conducted individually under the supervision of a trained research assistant, who provided standardized instructions at the beginning of each session and monitored participant safety throughout the exercise. To promote intervention fidelity, all research assistants followed the same written session script and received standardized training before the study. Throughout the entire session, participants wore a wrist device that continuously measured blood oxygen saturation and heart rate, enabling the monitoring of physiological changes associated with the mindful walking exercise. During the walk, they were instructed to maintain an upright but nonrigid posture, to synchronize movement with breathing, and to pay attention to the sensations of each step, feeling the contact of the feet with the ground and the shifting of body weight from heel to toe. When distractions or thoughts arose, participants were encouraged to gently acknowledge them and bring attention back to the experience of walking and breathing. They were also invited to notice environmental stimuli, such as colours, sounds, or objects, without evaluation, remaining fully present in the moment. Each session thus integrated mindful

breathing, sensory awareness, and conscious movement into a continuous meditative experience. Adverse events were monitored at each session through participant inquiry and observation by the research assistant during the mindful walking practice. Any discomfort, falls, near-falls, dizziness, pain, fatigue, shortness of breath, or other unexpected symptoms were to be recorded according to type, severity, timing, duration, action taken, and presumed relatedness to the intervention.

4. As stated before, the A2 phase, at the completion of the training program, and the A3 phase, one month following the conclusion of training, were all performed according to the same protocol as the A1 phase, except for the MoCA used to describe the cognitive function profile of the participants.

Statistical Analysis

Given the pilot nature of the study and the limited sample size (N = 13), no formal a priori power calculation was conducted. In pilot and feasibility studies, effect size estimates and variance parameters are often uncertain, and traditional power-based sample size determination may be unreliable or misleading (Button et al., 2013). Instead, the present analyses were designed to maximize the informational value of the available data and to generate preliminary estimates to inform the design of future controlled trials. A Bayesian analytic framework was adopted, as it allows for probabilistic estimation of parameters and explicit quantification of uncertainty, which is particularly suitable for small-sample longitudinal designs (Kruschke, 2015).

Given the longitudinal design (Pre, Post, Follow-up) and the small sample size (N=13) for the paired observations, a frequentist approach based on traditional rank tests (e.g., Wilcoxon Signed-Rank Test) might lack power and only provide a binary (reject/fail to reject) inference. To overcome these limitations and provide a more robust and complete probabilistic statement regarding the intervention's effect, all analyses were conducted using Bayesian Hierarchical Modeling (BHM), which is conceptually like a robust, non-parametric paired t-test. Specifically, for each psychological and quality-of-life outcome,

within-person changes were examined across three paired comparisons: Post vs. Pre, Follow-up (FU) vs. Pre, and FU vs. Post. The model was implemented using the PyMC library in Python (Abril-Pla et al., 2023). For each comparison, the difference score ($\Delta = T_n - T_{n-1}$; $T_n - T_{n-2}$) was modelled using a Student's t-distribution (instead of a normal distribution), thereby providing robustness against potential outliers and avoiding strong parametric assumptions, which is highly appropriate for psychometric data. Weakly informative priors (Normal(0, 10) for the mean difference, Half-Normal (10) for the standard deviation, and Exponential (1/29) + 1 for the degrees of freedom ($\mu\Delta$) were used. Inference was based on the 95% Highest Density Interval (HDI) of the posterior distribution for the mean difference ($\mu\Delta$). An effect was considered evident if the 95% HDI did not include zero, and results were further supported by the Posterior Probability (e.g., $P(\mu\Delta>0)$), which quantifies the evidence for a directional change (e.g., Kruschke, 2015; Kruschke & Liddell, 2018).

Consistent with the exploratory objectives of the study, these analyses were not intended to provide confirmatory tests of intervention efficacy. Rather, posterior estimates were used to evaluate whether observed patterns of change were compatible with meaningful within-person variation and to derive parameters (e.g., variability, pre–post correlations) relevant for planning future controlled research.

References

Abril-Pla, O., Andreani, V., Carroll, C., Dong, L., Fonnesbeck, C. J., Kochurov, M., ... & Zinkov, R. (2023). PyMC: a modern, and comprehensive probabilistic programming framework in Python. *PeerJ Computer Science*, *9*, e1516.

Bosco, A., Spano, G., Caffò, A. O., Lopez, A., Grattagliano, I., Saracino, G., Pinto, K., Hoogeveen, F., & Lancioni, G. E. (2017). Italians do it worse. Montreal Cognitive Assessment (MoCA) optimal cut-off scores for people with probable Alzheimer's disease and with probable cognitive impairment. *Aging Clinical and Experimental Research*, *29*(6), 1113–1120. https://doi.org/10.1007/s40520-017-0727-6.

Button, K. S., Ioannidis, J. P. A., Mokrysz, C., Nosek, B. A., Flint, J., Robinson, E. S. J., & Munafò, M. R. (2013). Power failure: Why small sample size undermines the reliability of neuroscience. *Nature Reviews Neuroscience*, *14*(5), 365–376. https://doi.org/10.1038/nrn3475

Gauthier, S., Reisberg, B., Zaudig, M., Petersen, R. C., Ritchie, K., Broich, K., Belleville, S., Brodaty, H., Bennett, D., Chertkow, H., Cummings, J. L., de Leon, M., Feldman, H., Ganguli, M., Hampel, H., Scheltens, P., Tierney, M. C., Whitehouse, P., & Winblad, B. (2006). Mild cognitive impairment. *The Lancet*, *367*(9518), 1262–1270. https://doi.org/10.1016/S0140-6736(06)68542-5

Gotink, R. A., Meijboom, R., Vernooij, M. W., Smits, M., & Hunink, M. M. (2016). 8-week mindfulness based stress reduction induces brain changes similar to traditional long-term meditation practice: A systematic review. *Brain and Cognition*, *108*, 32–41. https://doi.org/10.1016/j.bandc.2016.07.001

Hidayati, A. R., Gondodiputro, S., & Rahmiati, L. (2018). Elderly profile of quality of life using WHOQOL-BREF Indonesian version: A community-dwelling. *Althea Medical Journal*, *5*(2), 105–110. https://doi.org/10.15850/amj.v5n2.1354

Kruschke, J. K. (2015). *Doing Bayesian data analysis: A tutorial with R, JAGS, and Stan* (2nd ed.). Academic Press.

Kruschke, J. K., & Liddell, T. M. (2018). The Bayesian New Statistics: Hypothesis testing, estimation, meta-analysis, and power analysis from a Bayesian perspective. *Psychonomic bulletin & review*, *25*(1), 178-206.

Marshall, G. A., Rentz, D. M., Frey, M. T., Locascio, J. J., Johnson, K. A., Sperling, R. A., & Alzheimer's Disease Neuroimaging Initiative. (2011). Executive function and instrumental activities of daily living in mild cognitive impairment and Alzheimer's disease. *Alzheimer's & Dementia*, *7*(3), 300–308. https://doi.org/10.1016/j.jalz.2010.04.005

Pérès, K., Helmer, C., Amieva, H., Orgogozo, J. M., Rouch, I., Dartigues, J. F., & Barberger-Gateau, P. (2008). Natural history of decline in instrumental activities of daily living performance over the 10 years preceding the clinical diagnosis of dementia: A prospective population-based study. *Journal of the American Geriatrics Society*, *56*(1), 37–44. https://doi.org/10.1111/j.1532-5415.2007.01499.x

Pocklington, C., Gilbody, S., Manea, L., & McMillan, D. (2016). The diagnostic accuracy of brief versions of the Geriatric Depression Scale: A systematic review and meta-analysis. *International Journal of Geriatric Psychiatry*, *31*(8), 837–857. https://doi.org/10.1002/gps.4407

Silva, P. A. B., Soares, S. M., Santos, J. F. G., & Silva, L. B. (2014). Cut-off point for WHOQOL-bref as a measure of quality of life of older adults. *Revista de Saúde Pública*, *48*(3), 390–397. https://doi.org/10.1590/S0034-8910.2014048004912

Teut, M., Roesner, E. J., Ortiz, M., Reese, F., Binting, S., Roll, S., Fischer, H. F., Michalsen, A., Willich, S. N., & Brinkhaus, B. (2013). Mindful walking in psychologically distressed individuals: A randomized controlled trial. *Evidence-Based Complementary and Alternative Medicine*, *2013*, 489856. https://doi.org/10.1155/2013/489856
